# Critical Role of Arcuate Y4 Receptors and the Melanocortin System in Pancreatic Polypeptide-Induced Reduction in Food Intake in Mice

**DOI:** 10.1371/journal.pone.0008488

**Published:** 2009-12-30

**Authors:** Shu Lin, Yan-Chuan Shi, Ernie Yulyaningsih, Aygul Aljanova, Lei Zhang, Laurence Macia, Amy D. Nguyen, En-Ju Deborah Lin, Matthew J. During, Herbert Herzog, Amanda Sainsbury

**Affiliations:** 1 Neuroscience Program, Garvan Institute of Medical Research, St Vincent's Hospital, Sydney, New South Wales, Australia; 2 School of Medical Sciences, University of New South Wales, Sydney, New South Wales, Australia; 3 Faculty of Medicine, University of New South Wales, Sydney, New South Wales, Australia; 4 Cancer Genetics and Neuroscience Program, Department of Molecular Virology, Immunology and Medical Genetics, and the Comprehensive Cancer Center, Ohio State University, Columbus, Ohio, United States of America; AgroParisTech, France

## Abstract

**Background:**

Pancreatic polypeptide (PP) is a potent anti-obesity agent known to inhibit food intake in the absence of nausea, but the mechanism behind this process is unknown.

**Methodology/Principal Findings:**

Here we demonstrate that in response to i.p. injection of PP in wild type but not in Y4 receptor knockout mice, immunostaining for the neuronal activation marker c-Fos is induced specifically in neurons of the nucleus tractus solitarius and the area postrema in the brainstem, notably in cells also showing immunostaining for tyrosine hydroxylase. Importantly, strong c-Fos activation is also detected in the arcuate nucleus of the hypothalamus (ARC), particularly in neurons that co-express alpha melanocyte stimulating hormone (α-MSH), the anorexigenic product of the proopiomelanocortin (POMC) gene. Interestingly, other hypothalamic regions such as the paraventricular nucleus, the ventromedial nucleus and the lateral hypothalamic area also show c-Fos induction after PP injection. In addition to c-Fos activation, PP injection up-regulates POMC mRNA expression in the ARC as detected by *in situ* hybridization. These effects are a direct consequence of local Y4 signaling, since hypothalamus-specific conditional Y4 receptor knockout abolishes PP-induced ARC c-Fos activation and blocks the PP-induced increase in POMC mRNA expression. Additionally, the hypophagic effect of i.p. PP seen in wild type mice is completely absent in melanocortin 4 receptor knockout mice.

**Conclusions/Significance:**

Taken together, these findings show that PP reduces food intake predominantly via stimulation of the anorexigenic α-MSH signaling pathway, and that this effect is mediated by direct action on local Y4 receptors within the ARC, highlighting a potential novel avenue for the treatment of obesity.

## Introduction

The worldwide prevalence of obesity and type 2-diabetes are increasing at an alarming rate. Although reductions of only 5–10% body weight can significantly reduce the risk of obesity-associated co-morbidities such as type 2 diabetes [1], the majority of people who lose excess weight by lifestyle interventions regain the weight they lost within two years [2]. There is an urgent need for novel strategies to boost the effectiveness of lifestyle interventions for weight loss.

Recent interest has surged in the possible pharmaceutical use of ligands for Y receptors as anti-obesity agents. Y receptors (Y1, Y2, Y4, Y5 and y6) are a family of G-protein-coupled receptors with three endogenous ligands: the orexigenic neuropeptide Y (NPY) as well as the gut-derived satiety hormones peptide YY (PYY) and pancreatic polypeptide (PP). Much interest has focused on pharmacological antagonism of Y1 and Y5 receptors, due to their designation as ‘feeding receptors’ [3]. However, whereas Y1 receptor deficient mice exhibit reduced fasting-induced food intake, they develop late-onset obesity [4], [5]. Paradoxically, Y5 receptor knockout mice are hyperphagic and are not protected against leptin-deficiency-induced obesity [6], and a Y5 receptor antagonist failed to provide clinically meaningful effects in human weight loss trials [7]. Y2 receptors have also been flagged as potential targets for novel anti-obesity agents, since PYY3-36, an endogenous Y2-preferring ligand, reduces food intake in lean [8] and obese [9] humans and reduces body weight and adiposity after chronic administration to obese rodents [10], [11]. Like other gut-derived satiety hormones such as glucagon-like peptide-1, however, PYY3-36 has been shown to induce nausea or conditioned taste aversion at doses that reduce food intake [12], [13], [14]. This could limit the effectiveness of PYY3-36 or Y2-preferring agonists as treatments for obesity.

PP, like PYY and NPY, can act on all Y receptors, but PP has the highest affinity for Y4 receptors [15]. PP-like compounds are under development as potential anti-obesity agents, in light of the fact that short-term peripheral PP administration to lean people [16], [17] or to obese people with Prader Willi Syndrome [18], [19], [20] significantly reduces appetite and food intake. PP dose-dependently reduces food intake in freely fed and fasted mice, and this effect is entirely mediated through Y4 receptors since the effect was completely abolished in Y4 receptor knockout mice [21]. Low circulating levels of PP have been observed in obese people and in people with obesity caused by Prader Willi Syndrome as well as in congenitally obese rodents [19], [22], [23], [24]. Animal studies suggest that long-term administration of PP would lead to significant benefits in the treatment of obesity. Indeed, PP transgenic mice exhibit reductions in food intake, body weight and fat mass, as well as reduced gastric emptying [25], and long-term peripheral administration of PP to genetically obese *ob/ob* mice significantly reduces food intake and body weight while reducing gastric emptying and increasing energy expenditure [26]. Importantly from a clinical perspective, PP does not seem to induce nausea [16], [17]. This may be related to the fact that Y4 receptors are only expressed at significant levels in the hypothalamus and the brain stem [27], [28], which may allow for more specific effects with fewer side effects if Y4 receptors were targeted with anti-obesity treatments.

Y4 agonism with PP is thought to mediate effects on appetite and energy balance by actions within the brainstem, resulting in modulation of digestive processes such as gastric secretion, motility and emptying by modulating vagal cholinergic pathways [25], [29]. In keeping with vagally-mediated alterations in gut function, the ability of peripheral PP administration to decrease both efferent activity of the gastric vagal nerve as well as food intake was abolished in vagotomized rodents [26].

While Y4 agonism with PP in the brainstem is implicated in reducing food intake via indirect effects on gastrointestinal function, the hypothalamus also appears to be involved. Repeated administration of PP to mice over 24 hours significantly reduced mRNA expression of the orexigenic neuropeptide Y (NPY) and orexin in the hypothalamus [26]. However, it is not known in which hypothalamic nuclei and via which hypothalamic pathways Y4 agonism may reduce food intake. Y4 receptors are expressed in the arcuate nucleus of the hypothalamus (ARC) [27], [28], which is accessible to circulating factors such as PP [30]. The aim of this study was to determine the pathways subsequent to Y4 receptor activation via which PP reduces food intake, with particular focus on the early responses to Y4 agonism within the ARC. This was achieved with c-Fos immunostaining to determine which regions of the brain are activated at 30 minutes after PP injection, as well as double immunohistochemistry to determine which neurons are activated by PP, and *in situ* hybridization to determine acute effects of PP on expression of key hypothalamic regulators of energy homeostasis. Additionally, we used conditional deletion of Y4 receptors within the ARC to determine the specific involvement of these receptors in processes that regulate food intake in response to PP. Finally, we used germline knockout of molecules activated by Y4 agonism, in order to demonstrate the pathways through which Y4 receptor agonism induces its effects on the ARC and subsequently on food intake.

## Materials and Methods

### Ethics Statement and Animals

All research and animal care procedures were approved by the Garvan Institute / St Vincent's Hospital Animal Ethics Committee and the study was conducted in accordance with the Garvan Institute's guidelines for animal husbandry. Mice were housed under conditions of controlled temperature (22°C) and illumination (12-hour light cycle, lights on at 07:00 hours) with *ad libitum* access to water and normal chow (6% kilojoules from fat, 21% kilojoules from protein, 71% kilojoules from carbohydrate, 2.6 kilocalories/g, Gordon's Specialty Stock Feeds, Yanderra, NSW, Australia) unless otherwise stated. Adult male wild type and Y4 receptor knockout (*Y4* ^−/−^) or conditional Y4 receptor knockout (*Y4* ^lox/lox^) mice on a mixed C57/Bl6 - 129SvJ background, generated as described previously [31], as well as adult male melanocortin 4 receptor knockout (*MC4R* ^−/−^) mice on the same mixed background (The Jackson Laboratory, Bar Harbour, Maine USA) were used. The average body weight of wild type, Y4^−/−^ or *Y4* ^lox/lox^ mice used in these studies was 25.7±1.3 g with no significant difference among groups, and the average body weight of *MC4R* ^−/−^ mice was 30.9±1.2 g. Unless otherwise stated, all mice were fasted for 24 hours prior to administration of PP in order to maximize effects of PP on food intake and other parameters, as fasting has been shown to enhance the ability of other gut-derived satiety hormones to illicit significant hypophagic effects [32].

### Hypothalamus-Specific Y4 Receptor Deletion

Hypothalamus-specific Y4 receptor deletion was induced as previously described [33]. In brief, conditional *Y4* ^lox/lox^ mice were anesthetized with ketamine and xylazine at 100 mg/kg and 20 mg/kg (Parke Davis-Pfizer, Sydney, Australia and Bayer AG, Leverkusen, Germany, respectively), and unilaterally injected with a recombinant Cre-recombinase-expressing adeno-associated viral vector (AAV-Cre) using a stereotaxic frame (Kopf Instruments, Tujunga, CA, USA). The non-injected side of the hypothalamus was used as an internal control. As an additional control, additional *Y4* ^lox/lox^ mice were unilaterally injected with an empty adeno-associated viral vector (AAV-empty). Brain injection co-ordinates relative to bregma were posterior 1.94 mm, lateral ±0.3 mm, ventral 5.75 mm, corresponding to the ARC [34]. Cre-recombinase- or empty-expressing virus (10^−11^ Pfu in 1.5 µl) was injected unilaterally over 5 minutes using a 1.5 µl needle connected to a Hamilton Syringe (Hamilton Company, Reno, Nevada, USA). Mice were housed individually for the ensuring 28 days and allowed continuous *ad libitum* access to food and water (no fasting) prior to i.p. PP injection and subsequent determination of c-Fos-like immunoreactivity and POMC mRNA expression levels in the ARC as described below. In order to confirm *Y4* gene deletion in conditional knockout mice, genomic DNA was isolated from hypothalamic blocks from AAV-Cre injected and non-injected control mice as well as from the cerebellum of the same mice by standard technology. PCR was performed with primers (Primer A 5′-GCATCTGTACTGAGTGGC-3′ and Primer B 5′-ATCCTTCCTGCCTCTATG-3′) that only produce a product of 290 base pairs from DNA in which the Y4-receptor gene had been deleted. PCR conditions were 5 minutes denaturation at 95°C followed by 35 cycles of 1 minute at 95°C, 1 minute at 55°C and 40 seconds at 72°C.

### Determination of PP-Induced Changes in Food Intake

Wild type, *Y4* ^−/−^ or *MC4R* ^−/−^ animals were single housed with a paper towel on the bottom of the cage replacing fibrous bedding and allowed three days to acclimatize. Food was removed from the cage hopper 24 hours prior to experimentation. Animals either received PP (Bachem AG, Bubendorf, Switzerland) at 200, 300 or 500 µg/kg or saline vehicle (0.9% sodium chloride, 10 ml/kg) via intraperitoneal injection between 09:00–10:00 hours. Animals were given free access to chow diet directly following injection. Food intake was measured at 4, 8 and 24 hours following PP or vehicle administration and re-feeding. Food consumption was measured as the weight of food taken from the hopper minus the weight of food spilled on the cage floor. Data are presented as cumulative food intake, calculated as the sum of food consumed during the 24 hours directly following re-feeding. As the lowest dose of PP (200 µg/kg) and this dosing paradigm produced a significant reduction in food intake in wild type mice, this dose was used for all studies unless otherwise stated.

### Immunohistochemistry for Determination of PP-Induced Changes in Brain c-Fos or P-Erk1/2 Expression

Wild type and *Y4* ^−/−^ or unilaterally AAV-Cre-injected *Y4* ^lox/lox^ conditional knockout mice were injected with PP (i.p. 200 µg/kg body weight) or saline vehicle in a volume of 10 ml/kg between 10:00 to 12:00 hours. Mice used for determination of P-Erk1/2 immunoreactivity were allowed continuous *ad libitum* access to food prior to PP injection. At 30 or 90 minutes after i.p. injection for c-Fos immunoreactivity, or at 30 minutes after i.p. injection for P-Erk1/2 immunoreactivity, mice were deeply anaesthetized with ketamine / xylazine (100 mg/kg and 20 mg/kg from Parke Davis-Pfizer, Sydney, Australia and Bayer AG, Leverkusen, Germany, respectively) and perfused via the left ventricle and right carotid artery with 25 ml phosphate buffered saline following by ice-cold 4% paraformaldehyde made in phosphate buffered saline. This 30 minute time point was chosen because our previous experience with another molecule that regulates food intake, macrophage inhibitory cytokine-1 (MIC-1) [35], revealed that this 30 minute time point is important for determining specific neurons in the brain that are activated after peripheral administration of MIC-1. However, to more comprehensively investigate the activation of c-Fos in response to i.p. PP administration, we also looked at a 90 minute time point. Brains were immediately removed and placed in 4% paraformaldehyde for 30 minutes and then in phosphate buffered saline containing 30% sucrose in which they remained overnight. Coronal slices of 30 µm thickness were mounted on slides (Menzel-Glaser, Braunschweig, Germany) and washed in 1% H_2_O_2_ in 50% alcohol for 20 minutes to abolish endogenous peroxidase activity. Sections were incubated overnight at room temperature with the primary antibody, rabbit-anti-mouse c-Fos (Santa Cruz Biotechnology Inc, Santa Cruz, CA, USA) or rabbit-anti-mouse P-Erk1/2 (Cell Signaling Technology Inc, Danvers, MA, USA) diluted at 1∶4000 in phosphate buffered saline containing 0.1% Triton x-100. After three 10-minute washes in PBS-Triton, sections were incubated for 3 hours with the biotinylated secondary antibody (Sigma-Aldrich, St Louis, MO, USA), diluted 1∶250 in phosphate buffered saline. Sections were washed three times for 10 minutes each in phosphate buffered saline and then incubated with Avidin-Biotin-Peroxidase Vectastain® (Vector Laboratories, Burlingame, CA, USA) for 30 minutes at room temperature. Sections were rinsed in phosphate buffered saline and treated with diaminobenzidine (Dako, Carpinteria, CA, USA) for 5 minutes. Slides were rinsed in water and dehydrated through to xylene before cover slipping. Sections were visualized for c-Fos-like immunoreactivity or P-Erk1/2 immunoreactivity using a Zeiss Axiophot microscope equipped with the ProgRes digital camera (Carl Zeiss Imaging Solutions GmbH, Munich, Germany). Twelve sections from each mouse were counted by investigators blinded of the genotype and treatment of the samples for darkly stained nuclei representing c-Fos-like or P-Erk1/2 immunoreactivity within the brain nuclei of interest, which were defined according to the mouse brain atlas [34]. Data are represented as the average number of c-fos-like or P-Erk1/2 immunoreactive neurons per section in each nucleus, averaged over 4–6 mice per group.

### Double Labeling Studies for c-Fos Immunohistochemistry with TH, α-MSH or GAD65

Double labeling studies were performed to determine the chemical identity of neurons activated by PP injection, as indicated by c-Fos immunoreactivity. Six male wild type mice were used for each double labeling study. Twenty-four hour fasted wild type mice were injected with either PP (i.p. 200 µg/kg) or saline in a volume of 10 ml/kg between 10:00 to 12:00 hours. At 30 minutes after i.p. injection, mice were anesthetized and perfused and brains post-fixed as described above. Brains were frozen in mounting media (Tissue-Tek, ProScitech, Thuringowa, QLD, Australia) and 35 µm coronal sections were cut on a cryostat at −10°C. Brain sections were washed in 1% H_2_O_2_ in 50% alcohol for 20 minutes to abolish endogenous peroxidase activity.

The primary antibodies, rabbit-anti-mouse c-Fos (Santa Cruz Biotechnology Inc, Santa Cruz, CA, USA), sheep anti-mouse TH (Santa Cruz Biotechnology Inc) or sheep anti-mouse α-MSH (AB5087, Chemicon, Temecula, CA, USA), were diluted at 1∶4000, 1∶3000 or 1∶5000 respectively in phosphate buffered saline containing 0.1% Triton x-100 and then incubated with slide-mounted sections at room temperature overnight. After washing three times for 10 minutes each in phosphate-buffered saline-triton, sections were incubated for 3 hours at room temperature with goat anti-rabbit or donkey anti-sheep secondary antibodies conjugated to fluorophores (Molecular Probes® Alexa Fluor® 594 and Alexa Fluor® 488 from Invitrogen, Carlsbad, CA, USA), diluted 1∶250 in phosphate buffered saline. Sections were washed three times in phosphate buffered saline for 10 minutes each and fluorophore signals (red for c-Fos and green for TH or α-MSH) were visualized under a Zeiss Axioplan (Oberkochen, Germany) light microscope.

For c-Fos and GAD65 double immunostaining, primary antibodies, goat-anti-mouse c-Fos (Santa Cruz Biotechnology Inc, Santa Cruz, CA, USA) and rabbit-anti-mouse GAD65 (AB5082, Chemicon, Temecula, CA, USA) were diluted at 1∶4000 and 1∶2000 respectively in phosphate buffered saline containing 0.1% Triton x-100 and then incubated with slide-mounted sections at room temperature overnight. After washing three times for 10 minutes each in phosphate-buffered saline-triton, sections were incubated for 2 hours at room temperature with biotinylated anti-goat secondary antibody (Chemicon, Temecula, CA, USA) diluted 1∶250 in phosphate buffered saline. Sections were then washed three times in phosphate buffered saline for 10 minutes each and incubated for 30 minutes at room temperature with avidin-biotin-peroxidase (Sigma-Aldrich, St Louis, MO, USA). Sections were washed in phosphate buffered saline and treated with 0.05% diaminobenzidine tetrahydrochloride (Sigma-Aldrich, St Louis, MO, USA) and 0.007% hydrogen peroxide in the presence for 0.04% nickel ammonium sulphate to stain for c-Fos immunoreactivity. Section were then washed in phosphate buffered saline and incubated for 2 hours at room temperature with the biotinylated anti-rabbit secondary antibody (Sigma-Aldrich, St Louis, MO, USA) diluted 1∶250 in phosphate buffered saline. Sections were then washed three times for 10 minutes each in phosphate buffered saline and incubated for 30 minutes at room temperature with Avidin-Biotin-Peroxidase Vectastain® (Vector Laboratories, Burlingame, CA, USA). Sections were rinsed in phosphate buffered saline and treated with diaminobenzidine (Dako, Carpinteria, CA, USA) to detect GAD65 immunoreactivity. Slides were rinsed in water and dehydrated through to xylene before cover slipping.

To quantitate co-localization of c-Fos immunoreactivity with TH, α-MSH and GAD65 in the NTS, AP and ARC, the number of double-labeled neurons in each region was divided by the number of c-Fos-positive and the number of TH-, α-MSH-, or GAD65-positive neurons in each site. Stained neurons were counted in 3–4 distinct sections from both hemispheres.

### *In Situ* Hybridization for Quantification of POMC, GAD65 and NPY mRNA Expression

Ten 24 hour-fasted wild type mice and 8 *ad libitum*-fed unilaterally AAV-Cre-injected *Y4* ^lox/lox^ conditional knockout mice were injected with either PP (i.p. 200 µg/kg) or saline in a volume of 10 ml/kg between 10:00 to 12:00 hours. All mice were killed by cervical dislocation and decapitation 30 minutes after injection, and brains were immediately removed and frozen. Coronal sections, 20 µm thick, of fresh frozen brains were cut and thaw-mounted on charged slides and stored at −20°C until use. For radioactive *in situ* hybridisation, DNA oligonucleotides complementary to mouse POMC (5′-TGGCTGCTCTCCAGGCACCAGC-TCCACACATCTATGGAGG-3′), GAD65 (5′GCGTCCACAC TGCAAGGCCTTGTCTCCTGTGTCATAGGACAGGTC-3′) or NPY (5′-GAGGGTCAGTCCA CACAGCCCCATTCGCTTGTTACCTAGCAT-3′) were labeled with [^35^S] thio-dATP (Amersham Biosciences, Little Chalfont, Buckinghamshire, UK) using terminal deoxynucleotidyltransferase (Roche, Mannheim, Germany). The expression levels of POMC, GAD65 and NPY mRNA were evaluated by measuring silver grain densities over individual neurons from photo-emulsion-dipped sections, as described previously [33].

### Statistical Analyses

All data were assessed by factorial ANOVA followed by Fisher's or Contrasts post-hoc tests, using StatView version 4.5 or Super-ANOVA (Abacus Concepts Inc, CA, USA). For all statistical analyses, *P*<0.05 was accepted as being statistically significant.

## Results

### Y4 Agonism with PP Activates Neurons in Key Brainstem and Hypothalamic Nuclei Implicated in Energy Balance

**Figure 1:**
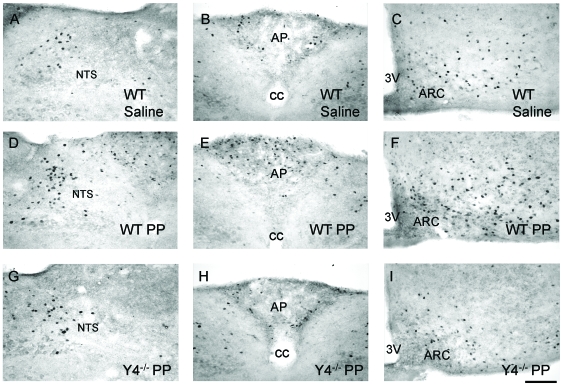
Pancreatic polypeptide (PP) injection induces a Y4 receptor-dependent increase in c-Fos immunoreactivity in the brainstem and hypothalamus. (A,B and C) Photomicrograph of brains from wild type (WT) mice showing c-Fos immunoreactivity at 30 minutes after i.p. injection of saline. (D, E and F) Brains from wild type mice showing c-Fos immunoreactivity at 30 minutes after i.p. injection of PP. (G, H and I) Brains from Y4 receptor knockout mice (*Y4* ^−/−^) showing c-Fos immunoreactivity at 30 minutes after i.p. injection of PP. Scale bar for all panels  = 40 µm. NTS, nucleus tractus solitarus; AP, area postrema; ARC, arcuate nucleus of the hypothalamus; 3V, third cerebral ventricle.

**Figure 2:**
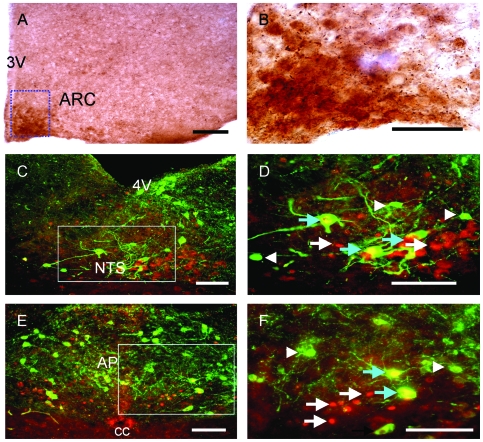
Immunoreactivity for phosphorylated extracellular signal-regulated kinases 1 and 2 (P-Erk1/2) in the hypothalamus and co-expression of c-Fos and tyrosine hydroxylase (TH) immunoreactivity in the brainstem after PP injection. (A) Brightfield micrograph showing P-Erk1/2 immunoreactivity in the arcuate nucleus of the hypothalamus (ARC) at 30 minutes after i.p. injection of PP in wild type mice. Scale bar = 200 µm. (B) Higher magnification of the boxed area from A. Scale bar  = 5 µm. (C, E) Fluorescence micrographs of the nucleus tractus solitarius (NTS) and area postrema (AP) respectively, 30 minutes after i.p. injection of PP. Scale bar  = 40 µm. (D, F) Higher magnifications of the NTS and AP, respectively, from the boxed areas in C and E. Scale bar  = 25 µm. White arrows indicate neurons positive for c-Fos immunoreactivity only (red fluorescence); white arrowheads indicate neurons positive for TH immunoreactivity only (green fluorescence); blue arrows indicate neurons double-labeled for c-Fos and TH. 4V, fourth cerebral ventricle; cc, central canal.

To determine which regions of the brain might be involved in mediating the PP-induced reduction in food intake and to identify the neuronal populations concerned, we tracked changes in expression of the neuronal activation marker, c-Fos, in response to i.p. PP injection. Significant increases in the number of c-Fos immunoreactive neurons were detected in the nucleus tractus solitarius (NTS) and the area postrema (AP) of the brainstem as well as in the ARC as early as 30 minutes after PP injection (Table 1, Figure 1). These c-Fos responses to PP were transient, because at 90 minutes post injection the levels of c-Fos positive neurons in the NTS, AP and ARC were no longer different from baseline levels (Table 1). However, other regions of the hypothalamus showed increased c-Fos labeling also at 90 minutes after PP injection, namely the paraventricular nucleus of the hypothalamus (PVN), the dorsomedial part of the ventromedial nucleus of the hypothalamus (VMHDM) and the lateral hypothalamic area (LHA, Table 1). Noteworthy is the fact that the level of c-Fos immunoreactivity in the PVN and LHA was significantly greater at 90 minutes than at 30 minutes after PP injection, suggesting that the heightened response in these nuclei at 90 minutes may include secondary responses to initial activation of other neurons in brain regions such as the brainstem or ARC. Saline injected control mice did not show any difference in c-Fos immunoreactivity at 90 minutes compared to 30 minutes in any brain area (data not shown). Consistent with a specific role of Y4 receptors in PP-induced neuronal activation, these increases in c-Fos immunoreactivity were not observed in the brains of Y4 receptor knockout mice after PP injection (Table 1, Figure 1). As further functional evidence that PP activates intracellular processes within the ARC, immunoreactivity for phosphorylated extracellular signal-regulated kinases 1 and 2 (P-Erk1/2), a marker of activation of intracellular signaling cascades affected by G-protein-coupled receptors, was strongly increased at 30 minutes after i.p. PP injection (Figure 2A+B).

**Table 1:** Number of c-Fos-like immunoreactive neurons in the brainstem and hypothalamus of wild type and Y4 receptor knockout (*Y4* ^−/−^) mice at 30 or 90 minutes after i.p. injection of saline or pancreatic polypeptide (PP).

Regions	Wild type Saline (30 min)	Wild type PP (30 min)	Wild type PP (90 min)	*Y4* ^−/−^ PP (30 min)
NTS	10±2	23±6*^#^	14±3	8±2
AP	3±1	26±5*^#^	5±2	3±1
ARC	9±2	38±5*^#^	13±3	8±1
PVN	15±2	42±6*^#^	60±16*	14±3
VMHDM	20±2	71±5*	67±6*	19±3
LHA	19±4	43±8*^#^	78±29*	17±3

Data are means ± SEM of 4–6 mice per groups.* P<0.05 versus saline-injected wild type mice; # P<0.05 versus PP-injected wild type mice (90 minutes). NTS: nucleus tractus solitarus; AP: area postrema; ARC: arcuate nucleus of the hypothalamus; PVN: paraventricular nucleus of the hypothalamus; VMHDM: dorsomedial part of the ventromedial nucleus of the hypothalamus; LHA: lateral hypothalamic area.

### Chemical Identity of Neurons Activated by Y4 Agonism with PP

To determine the chemical identity of neurons activated by i.p. PP injection and therefore gain insight into the mechanisms by which Y4 agonism with PP reduces food intake, we combined immunostaining for c-Fos with immunostaining for known key regulators of energy balance. In the NTS and AP of the brainstem, neurons containing c-Fos immunostaining at 30 minutes after PP injection also showed immunostaining for tyrosine hydroxylase (TH), the rate-limiting enzyme in the synthesis of catecholamines (Figure 2C–F). In fact, 52.1±6.1 and 46.4±5.0% of c-Fos positive neurons in the NTS and AP respectively were positive for TH immunoreactivity, and 85.2±4.5 and 66.1±11.5% of TH positive neurons in the NTS and AP respectively were positive for c-Fos immunoreactivity after PP injection (data are means ± SEM of 3–4 sections). These findings suggest that PP may induce part of its effects on food intake via catecholaminergic transmission from the NTS and AP to the hypothalamus, pathways known to exist between the hindbrain and hypothalamus [36]. Additionally, brainstem mechanisms have been shown to mediate effects of PP on gastrointestinal functions, which can in turn influence food intake indirectly [25], [26], [29]. In order to elucidate pathways by which PP may reduce food intake via direct effects on hypothalamic feeding centers, we investigated changes in the ARC in response to PP injection in further detail.

**Figure 3:**
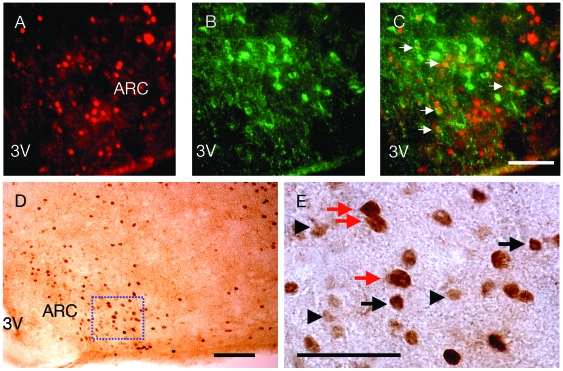
Pancreatic polypeptide (PP) injection induces c-Fos immunoreactivity in neurons positive for alpha melanocyte stimulating hormone (α-MSH) and glutamic acid decarboxylase 65 (GAD65) in the arcuate nucleus of the hypothalamus (ARC). (A) c-Fos immunoreactivity, (B) α-MSH immunoreactivity, and (C) overlay of c-Fos and α-MSH immunoreactivity in neurons as indicated by white arrows at 30 minutes after i.p. injection of PP. Sale bar for A–C  = 25 µm. (D) Brightfield micrograph showing c-Fos and GAD65 immunoreactivity at 30 minutes after i.p. injection of PP. Scale bar  = 200 µm. (E) Higher magnification of the boxed area from D. Black arrows indicate neurons positive for c-Fos immunoreactivity only. These neurons are darkly stained. Black arrowheads indicate neurons positive for GAD65 immunoreactivity only. These neurons are lightly stained. Red arrows indicate neurons double-labeled for c-Fos and GAD65. The double staining on these neurons makes these neurons appear larger than the neurons positive only for c-Fos or CAG65 immunoreactvity. Scale bar  = 5 µm. 3V, third cerebral ventricle.

In the ARC, neurons activated by PP (as shown by induction of c-Fos immunoreactivity at 30 minutes after i.p. injection) showed immunostaining for alpha melanocyte stimulating hormone (α-MSH), the anorexigenic product of the proopiomelanocortin (POMC) gene, as well as glutamic acid decarboxylase 65 (GAD65), a key enzyme in the synthesis of the neurotransmitter gamma-aminobutyric acid (GABA) (Figure 3). Indeed, 55.0±1.5 and 35.0±3.1% of ARC neurons that were positive for c-Fos after PP injection were also positive for α-MSH and GAD54, respectively. Conversely, 84.4±1.1 and 86.3±2.1% of α-MSH- and GAD54-positive neurons in the ARC were also positive for c-Fos immunoreactivity after PP injection (data are means ± SEM of 3–4 sections). These findings suggest a link between PP-induced hypophagia with α-MSH- and GABA-ergic signaling.

### Y4 Agonism with PP Stimulates POMC and Inhibits GAD65 Expression with No effect on NPY Expression in the ARC

**Figure 4:**
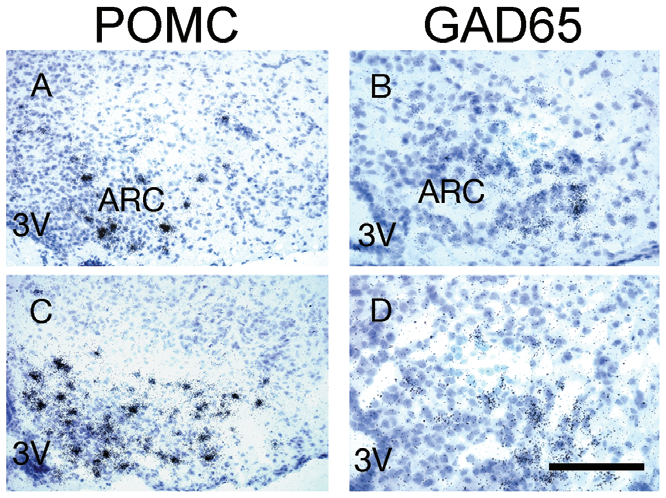
Effects of pancreatic polypeptide (PP) on the expression of proopiomelanocortin (POMC) and GAD65 mRNA in the arcuate nucleus of the hypothalamus (ARC). Emulsion-dipped autoradiographs showing POMC mRNA at 30 minutes after i.p. injection of (A) saline or (C) PP, orGAD65 mRNA at 30 minutes after i.p. injection of (B) saline or (D) PP. Scale bar for all panels  = 25 µm. 3V, third cerebral ventricle.

Our finding that PP activates c-Fos immunoreactivity in α-MSH-immunoreactive neurons, combined with the known role of ARC α-MSH to inhibit food intake and body weight gain [37], led us to hypothesize that the melanocortin system may be a key mediator of the anorectic effects of PP.We therefore investigated the expression of POMC in response to i.p. PP injection. Indeed, mice injected with PP showed a significant increase in ARC POMC mRNA levels relative to saline-injected controls at 30 minutes after injection (Table 2, Figure 4A+C). As ARC POMC neurons are negatively regulated by GABA-ergic neurons [38], [39], [40], [41], we also investigated the effect of PP on GAD65 expression. PP injection significantly reduced ARC GAD65 mRNA expression within 30 minutes compared to expression levels in saline-injected mice (Figure 4B+D, Table 2). Contrarily, PP had no effect on ARC NPY mRNA levels (Table 2), suggesting that PP-induced hypophagia is not mediated via changes in NPY expression. This is consistent with our observation that PP induced c-Fos immunoreactivity in cells in the lateral aspect of the ARC that preferentially express POMC but little NPY, whereas the dorsal aspect of the ARC, known to express NPY [42], showed little PP-induced c-Fos activation (Figure 1C+F).

**Table 2:** Expression of proopiomelanocortin (POMC), glutamic acid decarboxylase 65 (GAD65) and neuropeptide Y (NPY) mRNA in the arcuate nucleus of the hypothalamus at 30 minutes after i.p. injection of saline or pancreatic polypeptide (PP).

mRNA	saline	PP
POMC	100±12.4	268±12.0***
GAD65	100±17.3	60±9.2**
NPY	100±10.6	103±4.4

Data are means ± standard error of 5 wild type mice per group, with neuron labeling intensity expressed as a percentage of saline-injected controls. **, P<0.01; ***P<0.001 versus saline-injected controls.

### Direct Role of ARC Y4 Receptors in PP-Induced c-Fos Activation and Induction of POMC Expression

**Figure 5:**
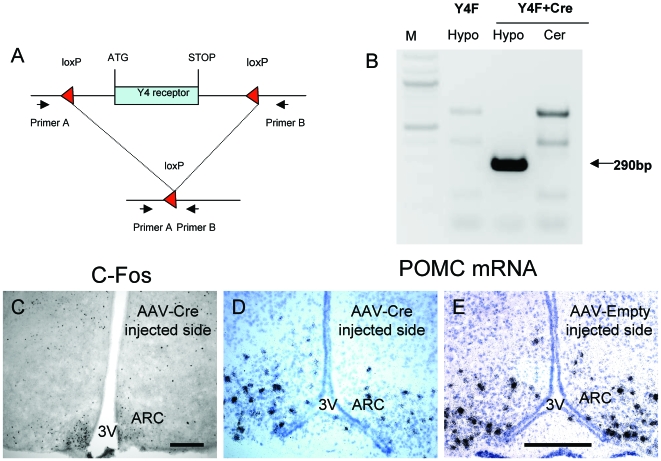
Conditional deletion of Y4 receptors in the arcuate nucleus of the hypothalamus (ARC) alters pancreatic polypeptide (PP)-induced c-Fos immunoreactivity and POMC mRNA expression. (A) Schematic representation of primer positions used for PCR verification of Y4 receptor gene knockout.The un-deleted gene is 3.4 kilobases, and after Cre-mediated Y4 receptor gene deletion a PCR product of 290 base pairs is produced. (B) Confirmation of Y4 receptor deletion (indicated by production of the 290 base pair PCR product) from DNA isolated from the hypothalamus (Hypo) but not from the cerebellum (Cer) of Y4 receptor conditional knockout (Y4F) mice injected with AAV-Cre recombinase into the ARC, or from mice not injected with AAV-Cre(Y4F Hypo). (C) Photomicrograph showing c-Fos immunoreactivity and (D+E) emulsion-dipped autoradiograph of POMC mRNA expression in the brain of a conditional Y4 receptor knockout mouse, 30 minutes after i.p. injection of PP. Mice received unilateral injection of (D) AAV-Cre or (E) AAV-empty 28 days prior to PP injection in order to induce local deletion of Y4 receptors by virally-delivered Cre-recombinase, the contra-lateral side (at left in C, D and E) was used as control. Scale bar  = 100 µm in C and 25 µm in D and E. 3V, third cerebral ventricle.

In order to determine the role of Y4 receptors specifically in the ARC in mediating PP-induced effects, we used conditional Y4 receptor knockout (*Y4* ^lox/lox^) mice which had been unilaterally injected into the ARC with a Cre-recombinase-expressing adeno-associated viral vector (AAV-Cre) in order to induce specific local Y4 receptor deletion, and then investigated c-Fos immunoreactivity and POMC mRNA expression in the ARC at 30 minutes after i.p. PP injection. Unilateral injection of AAV-Cre was chosen because by injecting only one side of the hypothalamus, one can directly use the contra-lateral side as an internal control, thereby avoiding inter-animal variations and demonstrating the direct consequences of lack of Y4 receptors on neuronal responses and gene expression. As an additional control, different animals were unilaterally injected with an empty adeno-associated viral vector (AAV-empty). Successful deletion of the Y4 receptor gene was confirmed by PCR of genomic DNA isolated from the hypothalamus of AAV-Cre-injected or non-injected mice using a primer set that only produces a product from DNA in which gene deletion has occurred (Figure 5A+B). As an internal negative control, DNA isolated from the cerebellum of the same AAV-Cre injected conditional knockout mice was used.

Results from this conditional knockout experiment show that in the non-AAV-Cre-injected contra-lateral side of the ARC, c-Fos immunoreactivity (Figure 5C) and POMC mRNA expression (Figure 5D) were increased after i.p. PP injection, whereas the AAV-Cre-injected side of the ARC, in which Y4 receptors had been deleted, showed markedly reduced c-Fos and POMC staining (19±2 versus 27±3 c-Fos positive neurons, and 81.4±1.2 versus 100±5.7 percent of POMC mRNA expression levels in the deleted versus non-deleted side of the ARC, data are means ± SEM of 8 mice, *P*<0.05). In mice that had been unilaterally injected with control virus (AAV-empty), no such reduction in POMC mRNA expression was seen in the injected versus the non-injected side of the hypothalamus after i.p. PP injection (Figure 5E). This result proves that Y4 receptors within the ARC are important mediators of local PP-induced c-Fos activation, and that PP action via Y4 signaling in the ARC controls POMC mRNA expression.

### Y4 Agonism with PP Reduces Food Intake via α-MSH Mediated Signaling

**Figure 6:**
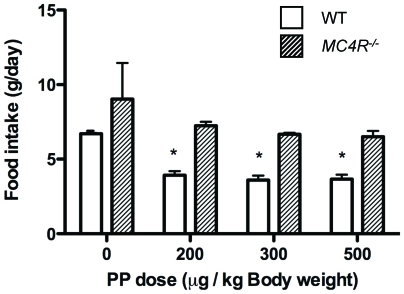
PP reduces food intake via a melanocortin 4 receptor-dependent pathway. Twenty four (24) hour food intake (g/day) in wild type (WT) and melanocortin 4 receptor knockout (*MC4R* ^−/−^) mice after i.p. injection of increasing doses of PP or saline vehicle. Data are means ± SEM of 6–8 mice per group. * P<0.05 versus vehicle-injected wild type mice or the comparison shown by horizontal bars.

To determine whether PP-induced c-Fos activation in α-MSH-immunoreactive neurons and up-regulation of POMC mRNA levels in the ARC in response to PP is functionally implicated in the anorectic effect of PP, we investigated PP-induced food intake in mice deficient in the melanocortin 4 receptor (MC4R), the receptor via which α-MSH induces its anorexigenic effects [43]. Interestingly, whereas i.p. injection of PP into wild type mice induced a significant reduction in food intake compared to saline-injected controls, no such effect of any of the three doses of PP used was seen after i.p. injection into MC4R knockout mice (Figure 6), demonstrating that PP mediates its anorectic effect via modulating MC4R pathways.

## Discussion

This study demonstrates that reduction in food intake induced by Y4 receptor agonism with PP is mediated by activation of the POMC / α-MSH signaling pathway, because PP-induced hypophagia is absent in *MC4R* ^−/−^ mice. Moreover, the ability of Y4 receptor agonism with PP to activate the ARC POMC / α-MSH system is mediated by Y4 receptors within the ARC, since conditional knockout of Y4 receptors specifically in this area abolishes PP-induced activation of c-Fos and induction of POMC expression in the ARC. Furthermore, double labeling revealed that the ARC neurons activated by peripheral PP contained immunoreactivity for α-MSH as well as GAD65, a key enzyme in the synthesis of GABA. Besides enhancing ARC mRNA expression of the precursor for α-MSH, POMC, peripheral PP injection significantly inhibits expression of GAD65 with no effect on ARC NPY mRNA expression.Taken together, these findings suggest that peripheral PP may act on Y4 receptors within the ARC to activate α-MSH- and GABA-ergic neurons and thereby stimulate POMC and inhibit GAD65 mRNA expression, ultimately increasing α-MSH action on MC4Rs and reducing food intake.

While it is clear that hypothalamic Y4 receptors (i.e. in the ARC) are required for PP-induced neuronal activation and stimulation of POMC mRNA expression, this effect could be mediated by direct and / or indirect effects on POMC neurons. Y4 receptors are known to be expressed in the ARC [28], but it is not known on which neurons (e.g. POMC or GABA-ergic neurons) they are expressed. Our data support the possibility that PP influences POMC neurons via indirect action on GABA-ergic neurons. Indeed, PP increased c-Fos immunoreactivity in GAD65-expressing neurons, but at the same time significantly reduced GAD65 mRNA expression. Whereas PP-induced activation of c-Fos immunoreactivity in GAD65-expressing neurons is most likely via activation of the mitogen-activated protein (MAP) kinase pathway by the βγ-subunits of the G-protein that is coupled to Y4 receptors, inhibition of GAD65 mRNA expression in response to PP probably involves Y4 receptor-initiated inhibition of cAMP production via α subunits of the Gi/o protein coupled to Y4 [44], [45], thereby reducing GABAergic neurotransmission onto POMC neurons [46]. GABA synthesized in the ARC is released within the ARC as well as in other brain regions to modulate neuronal circuits that control physiological responses such as food intake. For instance, GABA-ergic nerve terminals synapse with POMC-containing neurons in the ARC [38], [39], and GABA reduces mRNA expression of POMC and inhibits the release of α-MSH in the ARC [40], [41]. Albeit co-localization of Y4 receptors on ARC GABA-ergic neurons remains to be demonstrated, these findings raise the possibility that the action of PP on Y4 receptors on GABA-ergic neurons in the ARC could contribute to PP-induced hypophagia via inhibition of GABA-ergic inputs to POMC neurons, with a subsequent increase in POMC expression and α-MSH transmission.

These data also show that – unlike the melanocortin system and GAD65 expression – ARC NPY mRNA expression was not altered after PP injection. This is in keeping with our observation that PP injection induced c-Fos activity in the lateral (not dorsal) aspect of the ARC, a region known to express POMC but not NPY [42]. Previously it was shown that four i.p. injections of PP over 24 hours (a total dose more than double the single acute dose used presently) significantly reduced NPY mRNA expression in the whole hypothalamus as determined by real-time PCR [26]. Our current data suggest that any effect of PP on hypothalamic NPY expression is not a primary effect of PP action. In contrast, this work reveals a key role of the melanocortin system in mediating satiety in response to PP. Interestingly, not all gut satiety hormones act via the melanocortin system. For instance, whereas i.p. PYY3-36 injection in mice modestly activates POMC neurons [8], [47] and significantly increases POMC mRNA expression in the ARC [32], the hypophagic effect of PYY3-36 is just as apparent in *MC4R* ^−/−^ as in wild type mice [47]. Therefore, other pathways besides the melanocortin system – such as those that induce nausea or food aversion [12] – must be important in mediating PYY3-36-induced satiety. The specific involvement of the melanocortin system in PP-induced satiety may explain why PP has been shown to reduce food intake without any reports of concomitant nausea [16], [17]. Although this work focusses on the hypothalamic melanocortin system, potential influences of the brain stem POMC system cannot be ruled out as contributing to PP-induced reductions in food intake.

In addition to the brainstem (NTS and AP) and ARC, our data show that other brain regions are likely involved in mediating responses to peripheral PP, as indicated by the induction of c-Fos immunoreactivity in the PVN, VMHDM and the LHA after PP injection. Activation of these hypothalamic regions may contribute to the ability of PP to not only reduce food intake but also induce other physiological effects that could contribute to reduced adiposity after longer-term administration [25], [26], such as enhancing the activity of sympathetic nerves innervating brown adipose tissue and stimulating energy expenditure in genetically obese *ob/ob* mice [26] and reducing glucose-induced insulin release after acute administration to obese rodents [20]. For instance, the PVN is implicated in regulation of sympathetically-mediated thermogenesis in brown adipose tissue [48], and the VMH is implicated in regulation of insulin secretion [49]. In light of previous reports that Y4 agonism with PP mediates effects on appetite and energy balance by actions within the brainstem to modulate digestive processes via vagal cholinergic pathways [25], [29], the ability of Y4 receptor agonism with PP to influence multiple central and peripheral pathways that impact on energy homeostasis makes Y4 agonists attractive potential anti-obesity agents.

Taken together, these data show that peripherally-administered PP reduces food intake via mechanisms requiring Y4 receptor signalling within the ARC and downstream POMC / α-MSH / MC4R pathways. These findings not only elucidate critical biological pathways for feeding behaviour, they also have important implications for the clinical treatment of obesity. As Y4 receptors are only expressed in specific regions of the brain relative to other Y receptors [27], [28], an advantage of developing Y4-specific agonists as novel anti-obesity agents could be a greater specificity in reducing food intake with fewer side-effects such as nausea.

## References

[1] (1998) Clinical Guidelines on the Identification, Evaluation, and Treatment of Overweight and Obesity in Adults–The Evidence Report. National Institutes of Health.. Obes Res.

[2] Wing RR, Hill JO (2001). Successful weight loss maintenance.. Annual Review of Nutrition.

[3] Iyengar S, Li DL, Simmons RM (1999). Characterization of neuropeptide Y-induced feeding in mice: do Y1-Y6 receptor subtypes mediate feeding?. Journal of Pharmacology & Experimental Therapeutics.

[4] Pedrazzini T, Seydoux J, Kunstner P, Aubert JF, Grouzmann E (1998). Cardiovascular response, feeding behavior and locomotor activity in mice lacking the NPY Y1 receptor.. Nature Medicine.

[5] Kushi A, Sasai H, Koizumi H, Takeda N, Yokoyama M (1998). Obesity and mild hyperinsulinemia found in neuropeptide Y-Y1 receptor-deficient mice.. Proceedings of the National Academy of Sciences of the United States of America.

[6] Marsh DJ, Hollopeter G, Kafer KE, Palmiter RD (1998). Role of the Y5 neuropeptide Y receptor in feeding and obesity.. Nature Medicine.

[7] Erondu N, Wadden T, Gantz I, Musser B, Nguyen AM (2007). Effect of NPY5R antagonist MK-0557 on weight regain after very-low-calorie diet-induced weight loss.. Obesity (Silver Spring).

[8] Batterham RL, Cowley MA, Small CJ, Herzog H, Cohen MA (2002). Gut hormone PYY3-36 physiologically inhibits food intake.. Nature.

[9] Batterham RL, Cohen MA, Ellis SM, Le Roux CW, Withers DJ (2003). Inhibition of food intake in obese subjects by peptide YY3-36.. New England Journal of Medicine.

[10] Pittner R, Moore C, Bhavsar S, Gedulin B, Smith P (2004). Effects of PYY[3-36] in rodent models of diabetes and obesity.. International Journal of Obesity.

[11] Chelikani PK, Haver AC, Reeve JR, Keire DA, Reidelberger RD (2006). Daily, intermittent intravenous infusion of peptide YY(3-36) reduces daily food intake and adiposity in rats.. Am J Physiol Regul Integr Comp Physiol.

[12] Halatchev IG, Cone RD (2005). Peripheral administration of PYY3-36 produces conditioned taste aversion in mice.. Cell Metabolism.

[13] Parkinson JR, Chaudhri OB, Kuo YT, Field BC, Herlihy AH (2009). Differential patterns of neuronal activation in the brainstem and hypothalamus following peripheral injection of GLP-1, oxyntomodulin and lithium chloride in mice detected by manganese-enhanced magnetic resonance imaging (MEMRI).. Neuroimage.

[14] Gantz I, Erondu N, Mallick M, Musser B, Krishna R (2007). Efficacy and safety of intranasal peptide YY3-36 for weight reduction in obese adults.. J Clin Endocrinol Metab.

[15] Blomqvist AG, Herzog H (1997). Y-receptor subtypes-how many more?. Trends in Neurosciences.

[16] Batterham RL, Le Roux CW, Cohen MA, Park AJ, Ellis SM (2003). Pancreatic polypeptide reduces appetite and food intake in humans.. J Clin Endocrinol Metab.

[17] Jesudason DR, Monteiro MP, McGowan BM, Neary NM, Park AJ (2007). Low-dose pancreatic polypeptide inhibits food intake in man.. Br J Nutr.

[18] Berntson GG, Zipf WB, O'Dorisio TM, Hoffman JA, Chance RE (1993). Pancreatic polypeptide infusions reduce food intake in Prader-Willi syndrome.. Peptides.

[19] Glaser B, Zoghlin G, Pienta K, Vinik AI (1988). Pancreatic polypeptide response to secretin in obesity: effects of glucose intolerance.. Hormone and Metabolic Research.

[20] Gettys TW, Garcia R, Savage K, Whitcomb DC, Kanayama S (1991). Insulin-sparing effects of pancreatic polypeptide in congenitally obese rodents.. Pancreas.

[21] Balasubramaniam A, Mullins DE, Lin S, Zhai W, Tao Z (2006). Neuropeptide Y (NPY) Y4 receptor selective agonists based on NPY(32–36): development of an anorectic Y4 receptor selective agonist with picomolar affinity.. J Med Chem.

[22] Tomita T, Greeley G, Watt L, Doull V, Chance R (1989). Protein meal-stimulated pancreatic polypeptide secretion in Prader-Willi syndrome of adults.. Pancreas.

[23] Reinehr T, Enriori PJ, Harz K, Cowley MA, Roth CL (2006). Pancreatic polypeptide in obese children before and after weight loss.. Int J Obes (Lond).

[24] Jia BQ, Taylor IL (1984). Failure of pancreatic polypeptide release in congenitally obese mice.. Gastroenterology.

[25] Ueno N, Inui A, Iwamoto M, Kaga T, Asakawa A (1999). Decreased food intake and body weight in pancreatic polypeptide- overexpressing mice.. Gastroenterology.

[26] Asakawa A, Inui A, Yuzuriha H, Ueno N, Katsuura G (2003). Characterization of the effects of pancreatic polypeptide in the regulation of energy balance.. Gastroenterology.

[27] Parker RM, Herzog H (1999). Regional distribution of Y-receptor subtype mRNAs in rat brain.. European Journal of Neuroscience.

[28] Parker R, Herzog H (2000). Localization of Y-receptor subtype mRNAs in rat brain by digoxigenin labeled in situ hybridization.. Neuropeptide Y Protocols.

[29] McTigue DM, Hermann GE, Rogers RC (1997). Effect of pancreatic polypeptide on rat dorsal vagal complex neurons.. Journal of Physiology.

[30] Jobst EE, Enriori PJ, Cowley MA (2004). The electrophysiology of feeding circuits.. Trends Endocrinol Metab.

[31] Sainsbury A, Schwarzer C, Couzens M, Jenkins A, Oakes SR (2002). Y4 receptor knockout rescues fertility in ob/ob mice.. Genes and Development.

[32] Challis BG, Pinnock SB, Coll AP, Carter RN, Dickson SL (2003). Acute effects of PYY_3-36_ on food intake and hypothalamic neuropeptide expression in the mouse.. Biochemical and Biophysical Research Communications.

[33] Sainsbury A, Schwarzer C, Couzens M, Fitissov S, Furtinger S (2002). Important role of hypothalamic Y2 receptors in bodyweight regulation revealed in conditional knockout mice.. Proceedings of the National Academy of Sciences of the United States of America.

[34] Franklin KB, Paxinos G (1997). The mouse brain in stereotaxic coordinates..

[35] Johnen H, Lin S, Kuffner T, Brown DA, Tsai VW (2007). Tumor-induced anorexia and weight loss are mediated by the TGF-beta superfamily cytokine MIC-1.. Nat Med.

[36] Date Y, Shimbara T, Koda S, Toshinai K, Ida T (2006). Peripheral ghrelin transmits orexigenic signals through the noradrenergic pathway from the hindbrain to the hypothalamus.. Cell Metab.

[37] McMinn JE, Wilkinson CW, Havel PJ, Woods SC, Schwartz MW (2000). Effect of intracerebroventricular alpha-MSH on food intake, adiposity, c-Fos induction, and neuropeptide expression.. Am J Physiol Regul Integr Comp Physiol.

[38] Horvath TL, Naftolin F, Leranth C (1992). GABAergic and catecholaminergic innervation of mediobasal hypothalamic beta-endorphin cells projecting to the medial preoptic area.. Neuroscience.

[39] Cowley MA, Smart JL, Rubinstein M, Cerdan MG, Diano S (2001). Leptin activates anorexigenic POMC neurons through a neural network in the arcuate nucleus.. Nature.

[40] Vergoni AV, Bertolini A (2000). Role of melanocortins in the central control of feeding.. Eur J Pharmacol.

[41] Jegou S, Blasquez C, Delbende C, Bunel DT, Vaudry H (1993). Regulation of alpha-melanocyte-stimulating hormone release from hypothalamic neurons.. Ann N Y Acad Sci.

[42] Smith MS (1993). Lactation alters neuropeptide-Y and proopiomelanocortin gene expression in the arcuate nucleus of the rat.. Endocrinology.

[43] Ellacott KL, Cone RD (2004). The central melanocortin system and the integration of short- and long-term regulators of energy homeostasis.. Recent Prog Horm Res.

[44] Levi R, Seyedi N, Schaefer U, Estephan R, Mackins CJ (2007). Histamine H3-receptor signaling in cardiac sympathetic nerves: Identification of a novel MAPK-PLA2-COX-PGE2-EP3R pathway.. Biochem Pharmacol.

[45] Mahon MJ, Bonacci TM, Divieti P, Smrcka AV (2006). A docking site for G protein betagamma subunits on the parathyroid hormone 1 receptor supports signaling through multiple pathways.. Mol Endocrinol.

[46] Acuna-Goycolea C, Tamamaki N, Yanagawa Y, Obata K, van den Pol AN (2005). Mechanisms of neuropeptide Y, peptide YY, and pancreatic polypeptide inhibition of identified green fluorescent protein-expressing GABA neurons in the hypothalamic neuroendocrine arcuate nucleus.. J Neurosci.

[47] Halatchev IG, Ellacott KL, Fan W, Cone RD (2004). Peptide YY3-36 inhibits food intake in mice through a melanocortin-4-receptor-independent mechanism.. Endocrinology.

[48] Wang C, Bomberg E, Levine A, Billington C, Kotz CM (2007). Brain-derived neurotrophic factor in the ventromedial nucleus of the hypothalamus reduces energy intake.. Am J Physiol Regul Integr Comp Physiol.

[49] Inoue S, Nagase H, Satoh S, Saito M, Egawa M (1991). Role of the efferent and afferent vagus nerve in the development of ventromedial hypothalamic (VMH) obesity.. Brain Res Bull.

